# P-31 - TUBERCULOSIS MORTALITY AND CONTRIBUTING FACTORS IN SCOTLAND: 2000-2023

**DOI:** 10.1093/pubmed/fdag046.082

**Published:** 2026-07-09

**Authors:** Eisin McDonald, Melissa Llano, Hazel Henderson, Mr Ebrahim Ghaderi, Navneet Rai, Morris C Muzyamba

**Affiliations:** Public Health Scotland; Public Health Scotland; Public Health Scotland; Public Health Scotland; Public Health Scotland; Public Health Scotland

## Abstract

**Background:**

Tuberculosis (TB) is still one of the important reasons for death in the world. The goals for 2035 are reducing the incidence and mortality by 90% and 95%, respectively. Since TB mortality has historically been high in Scotland, and we aim to achieve these goals using evidence informed actions.

**Objectives:**

To evaluate the risk factors related to TB mortality while considering confounders.

**Methods:**

In this historical cohort, we included all notified TB cases from 2000 to 2023 in Scotland. A death was defined as a death from any cause during the treatment of TB. Logistic regression (enter method) was utilized for the multivariate analysis, with a p-value of less than 0.05 considered significant.

**Results:**

A total of 8,465 TB cases were identified during the study period, of which 945 resulted in death. In the multivariate model, mortality was significantly higher in individuals aged 45–64 years (OR: 4.05, CI: 2.68–6.24, *p* < 0.001) and ≥65 years (OR: 14.93, CI: 9.80–23.26, *p* < 0.001), compared with those younger than 44 years. Several comorbidities were significantly associated with increased mortality, including malignancy (OR: 4.23, CI: 2.77–6.45, *p* < 0.001), chronic kidney disease (OR: 2.37, CI: 1.24–4.47, *p* = 0.008), chronic lung disease (OR: 2.37, CI: 1.73–3.24, *p* < 0.001), immunocompromised status (OR: 2.46, CI: 1.68–3.55, *p* < 0.001), previous TB (OR: 1.75, CI: 1.12–2.68, *p* = 0.011), and alcohol misuse (OR: 2.44, CI: 1.68–3.54, *p* < 0.001).

**Conclusion:**

This analysis demonstrates that advanced age is a major determinant of TB-related mortality, alongside comorbidities such as malignancy, chronic conditions, immunosuppression, and alcohol misuse.

